# Granulosa Cell Tumor of the Ovary: A Retrospective Study of 31 Cases and a Review of the Literature

**DOI:** 10.1155/2018/4547892

**Published:** 2018-03-29

**Authors:** Manel Dridi, Nesrine Chraiet, Rim Batti, Mouna Ayadi, Amina Mokrani, Khedija Meddeb, Yosra Yahiaoui, Henda Raies, Amel Mezlini

**Affiliations:** Department of Medical Oncology, Salah Azaïz Institute, Faculty of Medicine of Tunis, Tunis El Manar University, Tunis, Tunisia

## Abstract

**Background:**

Adult granulosa cell tumors (AGCTs) are the most common sex cord-stromal tumors. Unlike epithelial ovarian tumors, they occur in young women and are usually detected at an early stage. The aim of this study was to report the clinical and pathological characteristics of AGCT patients and to identify the prognostic factors.

**Methods:**

All cases of AGCTs, treated at Salah Azaïz Institute between 1995 and 2010, were retrospectively included. Kaplan-Meier's statistical method was used to assess the relapse-free survival and the overall survival.

**Results:**

The final cohort included 31 patients with AGCT. The mean age was 53 years (35–73 years). Patients mainly presented with abdominal mass and/or pain (61%, *n* = 19). Mean tumor size was 20 cm. The majority of patients had a stage I disease (61%,  *n* = 19). Two among 3 patients with stage IV disease had liver metastasis. Mitotic index was low in 45% of cases (*n* = 14). Surgical treatment was optimal in almost all cases (90%, *n* = 28). The median follow-up time was 14 years (1–184 months). Ten patients relapsed (32%) with a median RFS of 8.4 years (6.8–9.9 years). Mean overall survival was 13 years (11–15 years). Stage I disease and low-to-intermediate mitotic index were associated with a better prognosis in univariate analysis (resp., *p* = 0.05 and *p* = 0.02) but were not independent prognostic factors.

**Conclusion:**

GCTs have a long natural history with common late relapses. Hence, long active follow-up is recommended. In Tunisian patients, hepatic metastases were more frequent than occidental series. The prognosis remains good and initial staging at diagnosis is an important prognostic factor.

## 1. Introduction

Granulosa cell tumors (GCTs) represent only 5% of all ovarian cancers. However, they are the most common subtype of ovarian sex-cord tumors (70%) [1].

They first were reported by Rokitanski in 1855 [2]. Although there is no consensus on the pathogenesis of these tumors, most investigators believe they originate from early ovarian mesenchyma as they are composed of granulosa cells, theca cells, and fibroblasts in different degrees [3].

Hyperoestrogenism reported in patients with GCT is related to tumor production of oestrogens, anti-Müllerian hormone, and inhibin B [4]. According to histological findings, two different subtypes of GCT were identified: adult (AGCT) and juvenile (JGCT). AGCTs are more frequent [5]. Surgery is the mainstay of treatment. Chemotherapy and/or radiotherapy are considered in patients with advanced stage or with unresectable recurrent disease [3]. In this study, we aimed to describe epidemiologic characteristics of AGCT in Tunisian population and then identify relapse and overall survival prognostic factors.

## 2. Methods

We conducted a retrospective single-center cohort study of all patients with AGCT diagnosed and treated in the Medical Oncology Department at Salah Azaïz Institute for cancer from 1995 to 2010. Quantitative variables were expressed as mean and median values. Qualitative variables are expressed as absolute and relative frequencies. Statistical analyses were performed using SPSS 20.0 software. Kaplan-Meier's statistical method was used to assess the recurrence-free survival and overall survival (95% confidence interval).

## 3. Results

A total of 31 women with a mean age of 53 years (35–73 years) were included in the study. 61% of cases presented with abdominal mass and/or abdominal pain in (*n* = 19). Postmenopausal bleeding was reported in 32% of cases (*n* = 10). Ultrasound imaging was performed in all cases and showed mainly cystic unilateral mass (80%  *n* = 25). Median tumor size was 20 cm (4–33 cm). Abnormally elevated levels of serum tumor marker CA-125 were reported in 42% of patients (*n* = 13). Inhibin B was not studied in any of our patients.

Histological features identified were micro/macrofollicles with cores “coffee bean” (74%), Call-Exner bodies (55%), and necrosis (22%). Mitotic index measured in only 22 patients was mainly low (64%). The staging breakdown was as follows: stage I represents 61% (stage IC: 58% of stage I), stage II 10%, stage III 19%, and stage IV 10%. Metastases locations were mainly liver (67%) and pleura (33%). The primary treatment was surgery in all cases. 28 patients (90%) underwent hysterectomy with bilateral salpingo-oophorectomy. Nonoptimal surgery was reported in 3 cases (10%). Intraoperative tumor rupture occurred in 5 patients (16%). Adjuvant treatment was chemotherapy followed by hormonal therapy in one woman (3%) and chemotherapy alone in 18 women (58%). No patient received adjuvant radiotherapy.

Adjuvant chemotherapy was a platinum-based regimen: cyclophosphamide-cisplatin in 13 patients (72%) and bleomycin-etoposide-cisplatin in 6 patients (28%).

Mean overall survival was 13 years (11–15 years). Overall survival at 10 years was 90%.

The median follow-up time was 14 years (1–184 months). Median RFS was 8.4 years (6.8–9.9 years). Relapses were reported in 10 patients (32%); among them, 6 had local recurrence (60%). Characteristics of these patients are shown in Table 1. All these women underwent surgery followed by platinum-based chemotherapy. Following univariate Cox regression modeling, only stage I disease and low-to-intermediate mitotic index were significantly associated with improved survival. In this stage, the 5-year OS was 98% versus 65% ( *p* = 0.04) and the median OS was 46.3 months versus 42 months (*p* = 0.01), respectively (Table 2). High mitotic index was associated with poor survival (42 months versus 46.3 months, *p* = 0.01). No independent prognostic factor was identified in the multivariate analysis.

## 4. Discussion

AGCT is a very rare tumor with a known good prognosis. In fact, only 31 patients were included in our study from 1995 to 2010, and this is the first published study conducted in a Tunisian population.

Since it is a rare disease, limited data are available [3]. Clinical findings of our population are comparable to the literature findings. AGCTs usually occur in menopausal or postmenopausal women (average age: 50–54 years) [6].

The most reported signs in the literature are abdominal pain and/or abdominal distension (30% to 50%) and hormonal event such as postmenopausal bleeding, amenorrhea, and intermenstrual bleeding [1]. The size usually reported in the literature is >10 cm (73.5%) but it can vary from a small nonpalpable lesion to large masses (3–24 cm) [5]. GCT presents at early stage in 81% of cases (stage I, 71%; stage II, 10%) and at late stage in 19% of cases (stage III, 11%; stage IV, 8%) [5]. In our study, the largest tumor size was 33 cm with stage III (19%) and large tumors were common in our study. Stage IV disease was comparable to literature (10%). Metastatic sites of GCTs pulmonary and skeletal metastases are uncommon; 15% of relapses occurred in retroperitoneum nodes [7]. Hepatic metastases are rare with an incidence of 5-6% of all GCT recurrences but authors think that these metastases are misdiagnosed as end-stage primary liver cancer [8]. We found higher rate of hepatic pleural metastasis and nodes compared to literature (67%, 33%, and 30%, resp.) because almost all cases were histologically confirmed.

The mainstay of treatment is a complete surgery (hysterectomy with bilateral salpingo-oophorectomy) with staging for early stage and debulking surgery for advanced stage or recurrent disease [5].

Fertility-preserving surgery with unilateral salpingo-oophorectomy is an option in young patients with stage IA GCT. Available data showed that there is not much difference in survival with a conservative approach when compared to the radical surgery (97% versus 98%, resp.). The 5-year and 10-year disease-specific survival was 97% and 94% [5].

Chemotherapy is recommended for patients with advanced stage and recurrent disease. In early stage GCT, only high risk patients (large tumors, tumors with high mitotic index, or ruptured tumors) should receive adjuvant chemotherapy [7].

The most used chemotherapy regimen is a BVP (bleomycin, vinblastine, and cisplatin) or a BEP regimen, which substitutes etoposide for vinblastine [9]. Hormonal therapies such as megestrol and LHRH (luteinizing hormone-releasing hormone) agonists seem to be efficient in relapsing patients [10]. In our study, only one patient was treated with hormonal therapy.

The majors factors suspected in a number of studies were age, tumor size, rupture of tumor, mitotic activity, nuclear atypia, aneuploidy (in 5–20% GCT), p53 overexpression, high Ki-67, and stage of the disease [7]. We noted that the disease stage was the most reported factor affecting survival in GCT patients. However, these studies are limited by their retrospective analysis, the small number of patients included, and the heterogeneity of the different populations. Wu et al. reported, in a large series of 100 cases of GCT, survival rates at 5 and 10 years of 98% and 96%, respectively, for stage I and 70% and 60%, respectively, for stage II [11]. Similar data were found by Park et al. as the 5-year and 10-year OS rates in early stage (stage I and II) disease were 99% and 90%, respectively, while in advanced stage (stages III and IV) they were 80% and 67%, respectively [4].

The same survival rates were found in our study with a significant prognostic value of stage as OS at 5 years was 98% in early stage and 65% in advanced stage (*p* = 0.04). Our study too presents the same limitations as it is retrospective and included a small number despite its spread over 15 years.

Patients whose tumors had a mitotic index < 4/10 HPF had a DFS at 80 months of 90% compared to 25% for patients with a higher mitotic index [7]. These data were consistent with our findings; high mitotic index was associated with worse OS (42 months versus 46.3 months, *p* = 0.01).

Discording data were reported regarding the prognostic value of age, tumor size, residual disease, and tumor rupture. In fact, Ayhan et al. found that patients aged below 60 years had better mean time of survival (154.6 versus 89.2 months, *p* = 0.015) [12]. Some studies showed that tumors larger than 10 cm had lower survival [3]. Thomakos et al. showed that increased tumor size by one cm was associated with 13% increase in recurrence risk. In our study, there was no impact of tumor size in recurrence (DFS at 5 years in tumor larger than 10 cm was 50% versus 65% in tumors less than 10 cm, *p* = 0.325) [3].

In Ranganath et al.'s study, median survival of GCT patients who underwent optimal cytoreduction was 60 months in contrast to 19 months for those who did not with a decrease in survival from 82% to 22% [13]. Tumor rupture was associated with a decrease in 25-year survival from 86% in patients with stage IA disease to 60% in patients with stage IC [7]. In our study, we did not find any impact on survival of postoperative residual disease and tumor rupture. OS at 5 years in patients with residual disease was 57% versus 70% in patients with optimal cytoreduction (*p* = 0.05). In case of tumor rupture, OS at 5 years was 68% versus 76% in other cases (*p* = 0.22).

In the light of Ala-Fossi et al.'s study findings, P53 mutation in GCT may be associated with poor prognosis [14].

GCTs have a tendency for late recurrence. The recurrence rate in our study was 32%, whereas it was 44% in Wu et al.'s study. In this letter, early relapses were significantly related to advanced stage [11]. The longest reported time to recurrence was 40 years [5]. In our study, median RFS was 8.4 years (6.8–9.9 years).

Local pelvic recurrence was reported in 70% of cases; only 9% of recurrences were abdominopelvic, 6% were retroperitoneal, 6% were pelvic and retroperitoneal, and 3% were abdominopelvic and retroperitoneal [15]. In our study, recurrences were mainly located in the pelvis (60%).

Multidisciplinary treatment approach usually consists of disease debulking followed by radiotherapy or chemotherapy and may prolong the DFS [5]. Brown et al. used bevacizumab in 8 patients with recurrent GCT. The response rate was 38% and median progression-free survival was 7.2 months [16].

## 5. Conclusion

Granulosa cell tumor is an uncommon ovary neoplasm. It is known for relapsing even years after a curative treatment. Hence, an active lifelong follow-up is recommended with clinical examination and tumor markers such as inhibin B [17]. Disease stage seems to be the only reliable prognostic factor. Knowing more about molecular pathogenesis of GCT may lead to identifying the place of targeted therapies in the disease management.

## Figures and Tables

**Table 1:** Characteristics of patients with recurrent disease.

	*N* = 10	100%
Age		
Mean (years)	52	—
Range	35–64	—
Stage		
Stage I	6	60%
Stages II–IV	4	40%
Tumor size		
Mean (cm)	20	—
Mitotic index		
Low	4	40%
Intermediate	1	10%
High	1	10%
Missing	1	40%
Type of resection		
Optimal	7	70%
Nonoptimal	3	30%
Tumor rupture		
Yes	1	10%
No	9	90%
Adjuvant chemotherapy		
Yes	5	50%
No	5	50%
Time to relapse		
Mean (years)	3	—
Site of relapse		
Pelvic	6	60%
Liver	1	10%
Abdominal nodes	3	30%

**Table 2:** Univariate analysis of overall survival.

	OS 5-year survival	*p*
Stage		
Stage I	98%	*0.04*
Stages II–IV	65%	
Tumor size (cm)		
<10	85%	0.06
≥10	78%	
Mitotic index		
Low	85%	*0.01*
Intermediate-high	60%	
Age (years)		
≤60	85%	0.9
>60	86%	
Nuclear atypia		
Yes	80%	0.3
no	87%	
Residual disease		
RO	70%	0.05
R1-2	57%	
Tumor rupture		
Yes	68%	0.22
No	76%	
